# Sustainable Synthesis of InP Quantum Dots via Rieke-Indium Reduction: Size Control and Ensemble Optical Properties

**DOI:** 10.1021/acs.inorgchem.5c02060

**Published:** 2025-08-18

**Authors:** Michael A. Müller, Stephen Schulz, Kai Schwedtmann, Thorben Starzynski, René Hübner, Alexander Eychmüller, Jan J. Weigand

**Affiliations:** † Chair of Inorganic Molecular Chemistry - Faculty of Chemistry and Food Chemistry, 9169 TU Dresden, 01062 Dresden, Germany; ‡ Chair of Physical Chemistry—Faculty of Chemistry and Food Chemistry, 9169 TU Dresden, 01062 Dresden, Germany; § Institute of Ion Beam Physics and Materials Research, 28414 Helmholtz-Zentrum Dresden-Rossendorf, Bautzner Landstrasse 400, 01328 Dresden, Germany

## Abstract

The aminophosphane-based synthesis of InP quantum dots (QDs) suffers from a significant drawback due to its low atomic efficiency, resulting in the production of three equiv of phosphonium salts as a byproduct for each equivalent of phosphorus atom incorporated into InP QDs. To address this limitation, we propose a novel synthetic approach for the fabrication of InP-QDs, utilizing Rieke-In (In*) as both an indium precursor and a simultaneous reducing agent for the aminophosphanes employed. This method not only enables a more efficient and sustainable synthesis of InP-QDs but also offers excellent size tunability, as demonstrated by the successful synthesis of green-emitting InP/GaP/ZnS-QDs. The observed remarkable emission quantum yield (QY) exceeding 40% and a narrow full-width at half-maximum (FWHM) of 50 nm further underscore the merits of this new synthetic route. The combination of good QY and precise size control makes this approach particularly intriguing and promising for various optoelectronic applications.

## Graphical abstract

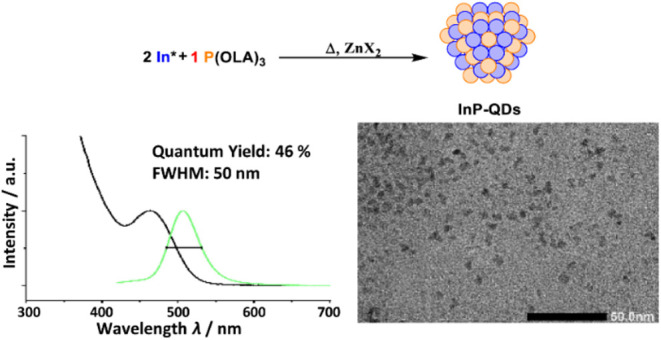

## Introduction

Colloidal quantum dots (QDs) have garnered significant research attention in recent years due to their size-dependent properties,^1–3^ which makes them highly promising for application in LED devices,^4^ bioimaging,^5^ catalysis,^6^ and various other fields.^7,8^ Early studies primarily focused on metal chalcogenides like CdSe^9^ and PbS.^10^ However, the toxic nature Cd- and Pb-based materials and the increasing environmental regulations necessitate the exploration of more environmentally benign alternatives.^11^ In this context, InP-based QDs have emerged as attractive candidates. They exhibit optical properties comparable to CdSe-QDs, positioning them as promising alternatives for optoelectronic devices and bioimaging application.^12^ Over the last two decades, significant progress has been made in the synthesis of InP-QDs, especially with the use of zinc salts to narrow particle size distribution. As a result, quantum yields approaching unity and fluorescence band widths at half-maximum (FWHM) below 40 nm have been achieved.^13^ Typically, P­(Me_3_Si)_3_ serves as the most commonly used P-precursor,^13–15^ known for its excellent and well-researched performance. However, its high cost, hazardous nature, and pyrophoric properties, along with specific handling requirements, have stimulated interest in seeking alternative precursors. Various alternatives have been proposed, including PH_3_,^16^ P_4_,^17^ (K/Na)_3_P,^18^ and aminophosphanes such as P­(NMe_2_)_3_.^19,20^ Among these alternatives, aminophosphanes stand out as a promising candidate,^21^ owing to their ease of handling, cost-effectiveness, and good stability. Prominent examples include the work of Reiss et al., whose synthetic approach is based on the use of Indium­(I) halide, which acts as both an indium source and a reducing agent for aminophosphine.^22^

In our contribution to this area, we introduced a long-time stable stock solution of P­(OLA)_3_ (OLA = oleylamine) as P-precursor, obviating the need for the previously employed in situ transamination reaction with aminophosphane systems.^23^ The competitiveness of aminophosphanes in InP-QDs synthesis was recently demonstrated with highly emitting QDs exhibiting a remarkable *QY* of 97% and FWHM of 37 nm.^24^ However, one significant drawback of this approach lies in the poor atom efficiency, as the incorporation of phosphorus atoms in InP-QD results in the production of three equiv of phosphonium salts as a couple-product (e.g., [P­(OLA)_4_] Cl; Scheme 1). This stems from the necessary redox reaction of the phosphorus atom, transforming from oxidation state +III in the precursor to −III in the product.^25,26^

**Scheme 1:**
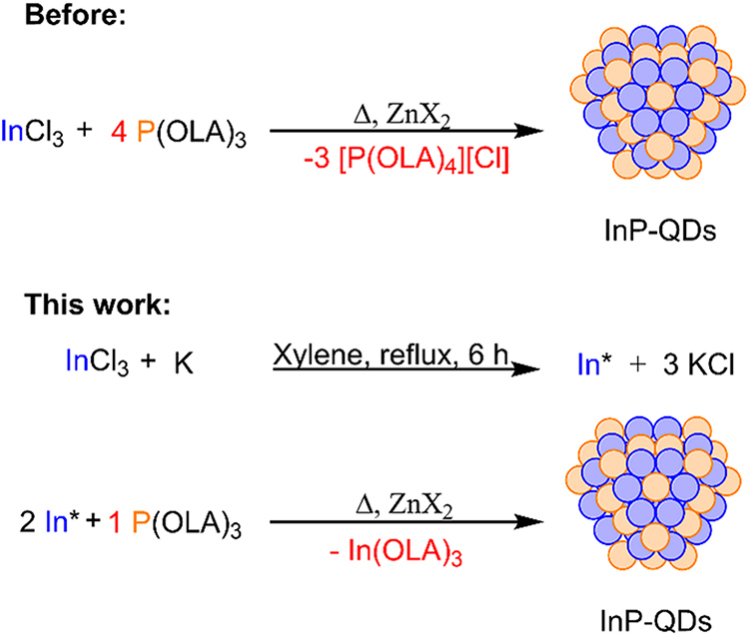
Synthesis of InP from Aminophosphane P­(OLA)_3_ with Rieke Indium (In*)

Additionally, the produced phosphonium salts may interfere with the shelling procedures.^23^ Therefore, the ideal reducing agent should possess properties that do not impede core growth or shelling and be economical, easy to remove, and recoverable, thereby significantly enhancing the application of aminophosphanes in InP-QD production. While indium­(I) halide salts have been a field of study recently and show promising results,^27^ their price is several times that of indium­(III) halide salts with comparable purity. Meanwhile, elemental Indium has emerged as a fitting solution and has been previously explored in combination with P­(Me_3_Si)_3_ for the production of InP-QDs.^28^ The combination of elemental indium with P­(Me_3_Si)_3_ did not deliver the desired results, as it hinders the size tunability of InP-QDs due to direct phosphidation of indium particles.

In this study, we introduce Indium nanoparticles (referred to as In*, Rieke-Indium) as a reducing agent for the reaction with aminophosphane P­(OLA)_3_, which effectively circumvented this problem. An additional advantage of our approach is the prevention of phosphonium salt formation (Scheme 1), significantly improving the atom efficiency and waste management, making it a more environmentally friendly synthesis of In-QDs. Furthermore, we demonstrate the remarkable size tunability of our system in the formation of InP-QDs, with optical properties such as FWHM and QY comparable to those reported for typical aminophosphane-based syntheses. Moreover, we successfully extended our approach to the synthesis of InP/GaP/ZnS QDs, further showcasing its versatility and potential for future optoelectronic applications.

## Results and Discussion

In our pursuit of increasing the atom efficiency and reducing waste generated during InP-QD synthesis via the aminophosphane route, we explored the potential of Rieke-indium (In*) as a potential reducing agent. Comparing the standard redox potential of In/In^3+^ (*E* = −0.34 V vs NHE) with the half-wave potential of the reduction of P­(OLA)_3_ (*E*_1/2_ = +0.17 V vs NHE, Figure S1, SI), elemental indium is expected to effectively reduce the aminophosphane precursor in a 1:2 ratio. This would result in the reduction of one “P^3+^” species to a “P^3–^”-equivalent for every two In^3+^-cations formed (Scheme 2). Notably, a 1:2 ratio of P:In is also the optimal stoichiometry for syntheses with P­(Me_3_Si)_3_ to obtain QDs with particularly low FWHM,^15^ a characteristic that we also confirmed for our approach (Figure S2, SI). Due to almost identical emission bandwidths of InP-based-QDs using In:P ratios of 1:1 and 1:0.5 as well as the equilibrated redox reaction in the latter case, higher In:P ratios were not investigated.

**Scheme 2:**
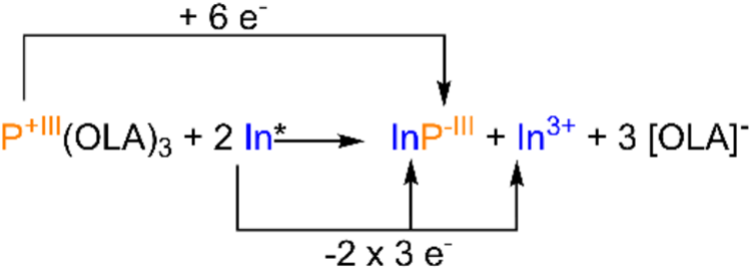
Proposed Redox Reaction for the Synthesis of InP-QDs Using Rieke-Indium (In*) as Reducing Agent

While elemental indium has a low melting point of 156 °C which would, in principle, allow its direct use at a typical reaction temperature of 200 °C for nanoparticle synthesis, the high surface tensions of metals prevent effective dispersion in the solvent. Thereby, the possible contact area to the phosphorus precursor in solution is vastly decreased compared to the one of nanoparticles, which limits the reaction speed and increases size dispersion and lowers the quantity of the obtained InP-QDs.^29^ As precursors, alkylindium derivatives were considered as a potential source of In particles.^28^ However, due to their challenging synthesis and high cost, and considering our goal to find a more cost-effective synthetic route (see Table S1, SI), we decided against their use. Furthermore, during QD synthesis, alkylindium derivatives tend to decompose, and the presence of surfactants such as trioctylphosphane (TOP) in the transformation process may potentially interfere with the subsequent QD growth. Therefore, we opted for an alternative approach that avoids the use of alkylindium derivatives and surfactants to ensure better control over the QD synthesis process.^28,30^

Instead, easy-to-prepare In* was used as an In precursor for InP-QD synthesis in this work. The synthesis of In* was successfully achieved by refluxing indium chloride with elemental potassium in xylene for 6 h under inert conditions (Scheme 1).^31^ As a byproduct of the reaction, KCl is formed but does not interfere with subsequent reactions and can be easily disposed of without posing any issues. Handling the In*-dispersion proved to be safer and more manageable compared to P­(SiMe_3_)_3_ as the dispersion was found to be nonpyrophoric. This characteristic makes it easier to work with during the experimental procedures. Subsequently, after removal of all volatiles, the formed In* was subjected to powder X-ray diffraction (pXRD) characterization. The diffraction pattern displayed the absence of typical reflexes for InCl_3_ and K, indicating a complete conversion of the reactants. The observed reflexes correspond only to In*, KCl, and Teflon,^32^ with the latter used for fixation and protection against air during measurement. Washing In* three times with methanol effectively removes KCl (see Figure 1a). It is important to note that while the challenges associated with using potassium as a reducing agent are acknowledged, the current focus of this paper is on the successful application of Rieke Indium (In*) for the formation of InP as a proof of principal, without specific emphasis on improving the synthesis of Rieke Indium itself. Addressing the improvement of the synthesis process of In* will be a subject of future research.

**Figure 1:**
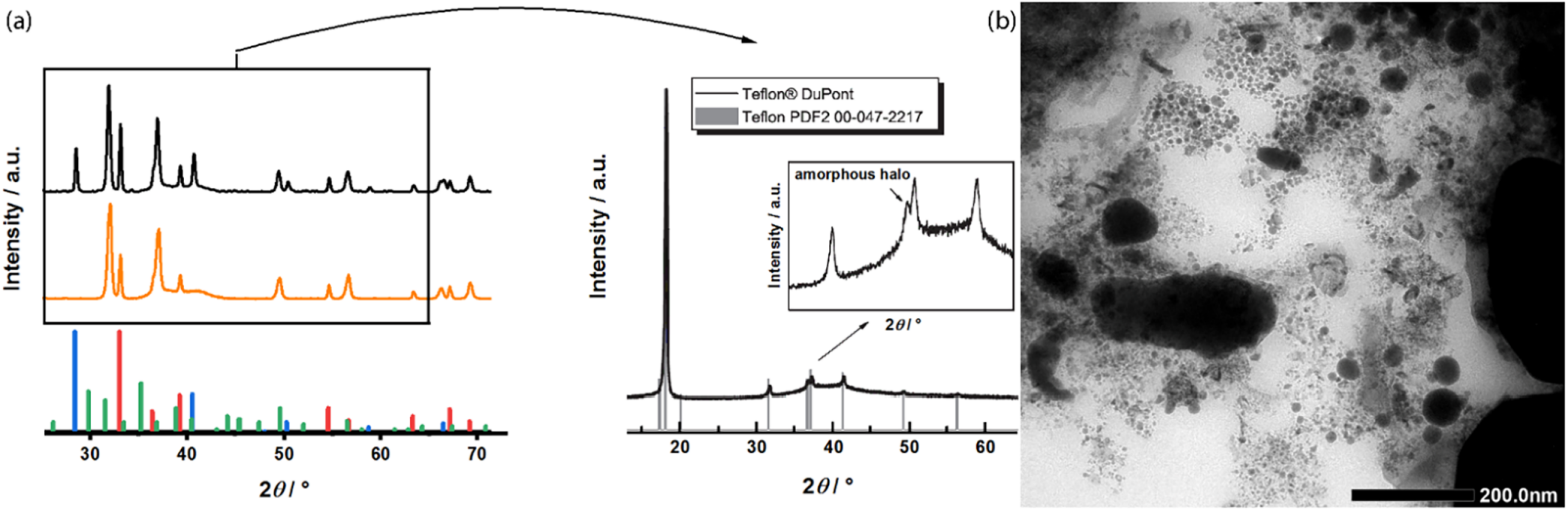
Powder XRD of as-synthesized In* (top) and In* washed three times with MeOH (bottom). The reflexes indicate the presence of elemental indium (red), KCl (blue), K (green), and Teflon (Powder XRD of Teflon, see right side, reproduced with permission from ref 31 Copyright © 2012 Elsevier B.V.). Notably, no green InCl_3_ is detected. Washing crude In* thrice with MeOH removes KCl efficiently (orange pattern). (b) TEM image of In* washed thrice with MeOH, revealing the morphology and characteristics of the synthesized material.

In* was found to crystallize in the body-centered tetragonal crystal structure with space group *I*4/mmm, consistent with the reference pattern for bulk material. For transmission electron microscopy (TEM) imaging, coprecipitated KCl was removed from a 1 mL sample of a 0.2 M In* solution through a series of washes with dry MeOH (3 × 25 mL). The TEM image of KCl-free In* in Figure 1b reveals a heterogeneous In* particle size distribution ranging from five nm to several hundred nm, likely attributed to particle agglomeration due to the absence of a surfactant shell.^33^ This is consistent with previous characterizations of In*.^34^ Despite this apparent polydispersity, we propose that the leaching or surface oxidation of In* gives rise to soluble In^3+^ species that serve as the actual precursor pool for InP QD formation. This would explain the narrow size distribution observed in the final QDs, despite the *hetero*geneity of the In* source. While finer In* powders appeared to give slightly higher QD yields in our hands, this observation remains qualitative and was not systematically investigated.

Interestingly, we discovered that it was not necessary to remove the coprecipitated KCl for subsequent reaction. Consequently, we utilized the as-produced In* without further purification, which was synthesized over 6 h in boiling xylene, for the synthesis of InP-QDs. This discrepancy between In* polydispersity and the observed size homogeneity in InP-QDs suggests a leaching-driven mechanism in which soluble In^3+^ species are formed in situ and serve as the reactive precursor pool. In* acts in a dual role as both an In precursor and a reducing agent in the heat-up synthesis conducted in octadecene (ODE). In this process, we employed Zn­(myristate)_2_ as the zinc salt and P­(OLA)_3_ in the ODE as phosphorus precursor, unless otherwise specified (synthetic procedure 1). It is intriguing to observe that the use of In* as an Indium precursor in the synthetic procedure leads to a significant improvement in the optical properties of the InP-QDs compared to InCl_3_. Specifically, it results in a considerable narrowing of the FWHM from 65 to 47 nm and a blue shift of the emission from 591 to 488 nm (Figure 2a). The authors attribute the narrowing of the FWHM to a higher reaction rate between In* and P­(OLA)_3_ compared to that between InCl_3_ and P­(OLA)_3_. This assumption is supported by the observed blue shift which according to the LaMer model for crystal growth is a result of faster precursor depletion. It is known that the latter reaction rate is slower than optimal for the formation of InP-QDs. Additionally, following the work up routine used for InP-QDs syntheses on the In* stock solution and measuring an ultraviolet–visible (UV/vis) spectrum of the supernatant, no absorption was observed. This indicates that the work up procedure assures that no residual In* affects the UV/vis spectra. Another method employed for the synthesis of quantum dots is the hot-injection method (synthetic procedure 2), in which the same amounts and reagents are used as for the heat-up procedure. However, instead of adding the precursors before the heating, P­(OLA)_3_ is introduced in the hot indium precursor solution at a reaction temperature of 200 °C. This approach enables more reliable monitoring of QD formation using ^31^P nuclear magnetic resonance (NMR) spectroscopy. In typical InP-QD syntheses using indium salts and an aminophosphane, the phosphonium salt [P­(OLA)_4_]­X is formed via disproportionation of P­(OLA)_3_ (X = Cl, Br, I, OTf, myristate, OAc). However, for synthesis procedure 2 in ODE, we observed that the phosphonium salt [P­(OLA)_4_]­X is generated only as a minor byproduct during the reaction, regardless of the reaction time as shown in Figure S3 for the case when Zn­(myristate)_2_ is used as zinc source. This indicates that the reported reaction follows a distinct mechanism. Notably, when synthesis procedure 2 is carried out in oleylamine as solvent, there is an increased formation of the byproduct [P­(OLA)_4_]­X, and the QD yield is lower. This emphasizes the influence of solvent selection on the reaction outcome. While a precise comparison is not possible, especially in oleylamine-free synthesis, the amount of isolated InP-QDs compared to the amount of phosphonium salt observed in ^31^P NMR at 33.14 ppm indicates that the disproportionation reaction of P­(OLA)_3_ is a negligible side reaction (Figure S3).

**Figure 2:**
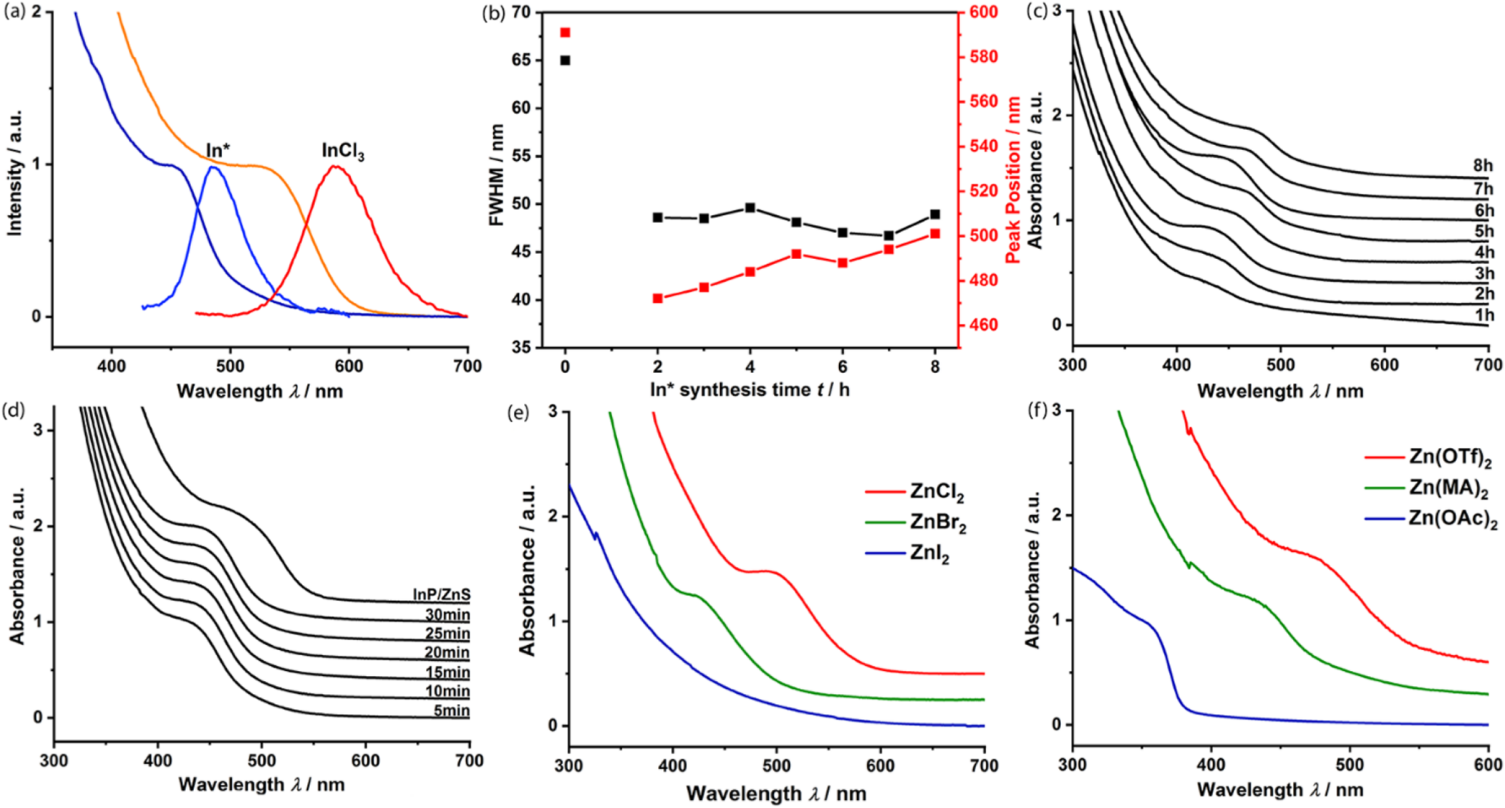
(a) Comparison of UV/vis absorption and fluorescence spectra of InP-QDs synthesized using InCl_3_ (red and orange lines) and In* (dark and light blue lines); (b) diagram depicting the FWHM and peak positions of bare InP-QDs synthesized using In* obtained after various synthesis times; (c) UV/vis absorption spectra measured after 30 min of core growth of InP synthesis, using In* obtained after various synthesis times; (d) UV/vis spectra of InP before and after ZnS shelling, In* obtained after 6h of reaction; (e) UV/vis spectra of InP synthesized using In* in the presence of zinc halogenides; (f) UV/vis absorption spectra of InP-QDs synthesized using In* in the presence of Zn­(OTf)_2_, Zn­(myristate)_2_ (Zn­(MA)_2_), and Zn­(OAc)_2_.

In oleylamine, both reaction routes appear to compete with each other. To investigate this further, we conducted a subsequent step in which we washed freshly prepared In*, obtained after 6 h of reaction time, three times with methanol (3x 25 mL) before proceeding with the synthesis procedure 1. This step aimed to confirm that the reaction was not influenced by any residual InCl_3_, as methanol is known to dissolve InCl_3_ and KCl. When InCl_3_- and KCl-free In* was used for the heating reaction, the formation of InP-QD was successful. However, we did observe a slight deterioration in the optical properties, which may be attributed to the formation of indium alcoholates during the washing process. Specifically, the FWHM increased from 47 to 59 nm, while the emission wavelength remained constant (Figure S4, SI). After successfully obtaining InP-QDs with narrower emission bands by omitting the methanol washing step, we continued using the in situ synthesized In* for our reactions. We conducted experiments by reacting InCl_3_ with potassium over varying periods, up to nine h, to form In* and then used different proportions of the in situ formed In* for InP-QD synthesis according to procedure 1. Surprisingly, we observed no significant change in the FWHM when using In* fractions that had been reacted for more than 2 h. However, a slight red shift in the peak positions of the luminescence bands was noticed over time (Figure 2b).

We further explored the impact of reaction time by performing a data point at 0 h of In* synthesis time. For this, the In* reaction setup was executed as usual, but instead of heating the reaction mixture of InCl_3_, potassium, and xylene to the refluxing temperature, the mixture was stirred for 5 min at room temperature. Subsequently, the mixture was utilized as the In*-dispersion in the synthesis of InP-QDs. Remarkably, the InP-QDs prepared using the In* fraction synthesized over 1 h did not exhibit fluorescence and were not successfully shelled. Furthermore, after 9 h of In* synthesis time, precipitation of the In* occurred. When a ZnS-shell was applied to these InP-QDs using zinc diethyldithiocarbamate as a single-source precursor (Figure S5, SI), we observed that the same trends in FWHM and peak position were maintained for the InP/ZnS core/shell system, reaffirming the consistency of our approach. We also investigated the optimal synthesis time for In* to achieve the best InP-QD properties. The UV/vis spectra of InP-QDs prepared with In* synthesized for up to 8 h are shown in Figure 2c. We found that the optimal properties of InP-QDs were obtained in reactions using In* synthesized over 6 h. This reaction time avoids the influence of residual InCl_3_ impurities and the agglomeration and precipitation of indium particles that occurs when In* is synthesized for more than 8 h.

To understand the kinetics of InP-QD core growth, we performed time-resolved measurements by measuring a reaction aliquot every 5 min after injection of P­(OLA)_3_ into the In*-containing reaction mixture for a total of 30 min of InP-QD core growth. We observed no shift in absorption characteristics during core growth, indicating that no particle growth occurred. This suggests rapid depletion of precursors, similar to observations with In salts and (Me_3_Si)_3_P as reagents.^35^ Interestingly, we found that the amount of InP-QDs produced is inversely proportional to the size of the In* particle at the start of the reaction. Initial experiments with macroscopic indium chips yielded little material, but its optical properties were identical with those synthesized from smaller In* particles. Therefore, obtaining smaller In* particles is crucial to maximize the reaction yield. Contrary to our expectations, increasing the equivalents of P­(OLA)_3_ used did not increase the amount of InP-QDs obtained. This underscores that the typical disproportionation reaction of the P-precursor is not responsible for the InP-QD formation. On the contrary, large amounts of P­(OLA)_3_ in the reaction had detrimental effects on QD properties, as evidenced by comparing UV/vis spectra and FWHM values of the emission band of InP-QDs obtained with different amounts of P­(OLA)_3_ (Figure S2). A significant increase in FWHM, accompanied by a blue shift, was observed with increasing amounts of utilized P­(OLA)_3_. The FWHM of unshelled InP-QDs gradually increased from 47 nm for 0.5 eq. of P­(OLA)_3_ to over 100 nm when 4 eq. of P­(OLA)_3_ were used, indicating the importance of controlling the amount of P­(OLA)_3_ for obtaining desired optical properties and cost-effectiveness. A blue shift in the emission wavelength was observed from 488 to 455 nm when using 0.5 and 4 equiv of P­(OLA)_3_, respectively. The same trends were observed for the ZnS-shelled QDs. As a similar blue shift has been reported for the reaction between In-salts and progressively larger amounts of aminophosphanes,^36^ our observations are attributed to a higher nucleation rate, limiting the subsequent QD growth and resulting in smaller-sized QDs and a higher FWHM due to Ostwald ripening.^37^

Regarding the formation mechanism of InP-QDs via our approach, two factors suggest a leaching/dissolution pathway rather than a direct conversion of In* particles via phosphidation. First, in the reaction of elemental In-NCs with P­(Me_3_Si)_3_, a direct conversion of homogeneously size-distributed In-NCs to InP-QDs was observed along with a size increase of 15–20%.^28^ However, in our approach, due to the broad size-distribution of In* as seen in TEM images, a different reaction mechanism must take place. If the phosphidation pathway had occurred in the described reaction, then we would have obtained an extremely broad size distribution for InP-QDs. However, this is not observed in either TEM images or UV/vis spectra. Second, the InP-QD ensembles obtained by our group exhibit tunable emission maxima and a narrow FWHM. The correlation between particle size and optical properties through the quantum confinement effect leads to wavelength shifts in the absorption and emission spectra, which cannot be explained by the phosphidation of broadly size-distributed In*.

To explore the size tunability of InP-QDs, we used various zinc salts (ZnCl_2_, ZnBr_2_, ZnI_2_, Zn­(OTf)_2_, Zn­(myristate)_2_, and Zn­(OAc)_2_) separately in hot injection reactions (synthesis procedure 2) to obtain UV/vis spectra of InP-QDs with differently shifted excitonic features. Similar to the synthesis using indium salts,^38^ we observed blue-shifted UV/vis excitonic features and thus, size tunability when using zinc salts with heavier halide anions (Figure 2e). The presence of different zinc salts, including halides, governs the particle size of InP-QDs during the synthesis of In-salts with aminophosphanes. This is attributed to the role of halide ions or other organic surfactants in quantum dot surface chemistry and the reactivity of phosphorus precursors.^38–40^ Using ZnCl_2_, we obtained InP and InP/ZnS-QDs with an emission maximum of 553 and 558 nm respectively, demonstrating the applicability of the approach also for the synthesis of QDs with longer wavelengths (Figure S6, SI). Size tunability was also observed when using Zn­(OAc)_2_, Zn­(myristate)_2_, and Zn­(OTf)_2_ (Figure 2f). Consequently, the dissolution and leaching of In-atoms or In^3+^-ions from In* must be a preliminary step in our InP-QD synthesis to obtain In-monomers necessary for nucleation and subsequent particle growth. Otherwise, no differing particle sizes would be observed. Notably, the InP-QDs with the narrowest emission band were obtained when using 2 equiv of Zn­(myristate)_2_ in the synthesis, achieving an impressive FWHM as low as 44 nm at an emission wavelength of 486 nm prior to shelling in the heat-up reaction. However, despite our efforts, we were unable to precipitate the unshelled InP-QDs from the oil we obtained after workup, which consequently prevented the acquisition of a pXRD pattern. Nevertheless, our synthesis with subsequent shelling produced InP/ZnS-QDs with a FWHM of 51 nm at 520 nm emission wavelength and an emission quantum yield QY < 10% (Figure S7, SI).

To further improve the quantum yield, InP-QDs were shelled with an GaP/ZnS multishell, following the recently reported synthesis of InP/GaP/ZnS-QDs via the aminophosphane route.^41^ In contrast to the multistep process required for obtaining InP/GaP-QDs in that study, we adopted a simple one-pot procedure to directly obtain InP/GaP-QDs, which were subsequently coated with a ZnS shell. When precipitation of InP/GaP and InP/GaP/ZnS-QDs was required, e. g., for TEM and pXRD measurements, the reaction was conducted in hexadecane (synthesis procedure 1a), while octadecane was used otherwise (synthesis procedure 1). Figure 3a shows the UV/vis spectra of the synthesized InP/GaP-QDs, where rapid depletion of the precursors is again observed, indicating efficient QD formation. Furthermore, the excitonic feature is found to be narrower than that of InP-QDs, indicating improved optical properties for potential applications. The pXRD patterns indicate minor incorporation of Ga or the formation of a thin GaP-shell in InP/GaP/ZnS-QDs (Figure 3b). TEM images display particles with an average size of 4.9 ± 1.2 nm (Figures 3c and S8). The pXRD reflexes exhibit a strong shift due to the applied ZnS shell (Figure 4b[Fig fig4],c). Further characterization using HAADF-STEM with energy-dispersive X-ray spectroscopy (EDXS) analysis on the fully shelled InP/GaP/ZnS-QDs revealed the formation of two different particle species. The first species lacked a ZnS-shell and mainly consisted of In, P, and Ga. The second species featured a small InP core with a thick ZnS-shell, and no incorporation of potassium from the In* stock solution was observed in either species. These findings strongly suggest the presence of two distinct populations rather than a uniform core–shell architecture. This aligns with the results of XRD and EDXS analyses, which point to a statistical mixture of InP-rich and GaP-containing particles. Accordingly, the observed emission characteristics arise from an ensemble of structurally distinct particles rather than a single, well-defined InP/GaP/ZnS core–shell architecture. While this may limit their immediate applicability in optoelectronics, the approach provides a scalable route toward high-quality emissive materials with promising properties. While this diverges from the idealized core–shell scenario, the resulting ensemble nonetheless exhibits favorable photophysical properties and demonstrates the potential of this synthetic strategy for accessing high-performance, heterostructure-like QDs by simple and scalable means. Optical characterization showed no fluorescence of InP or GaP before shelling. However, shelling with ZnS significantly increased the QY to 46% with a FWHM of 50 nm at an emission wavelength of 510 nm (Figure 4a), indicating that particles with a thick ZnS-shell contributed to the optical properties of the QD ensemble. This ZnS-shelling induced red shift is consistent with prior literature reports and attributed to a less-confined exciton.^38^ Repeatability of the synthesis is depicted in the UV/vis and PL spectra of three different experiments (Figure S9). Comparing the results with the more commonly used InP/ZnSeS/ZnS-core/shell/shell system, we observed a QY of 42% and a FWHM of 62 nm at an emission wavelength of 523 nm (Figure S10) using a modified literature procedure.^42^

**Figure 3:**
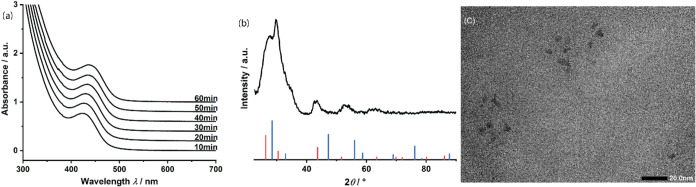
(a) UV/vis absorption spectra of InP/GaP-QDs at different growth times; (b) pXRD pattern of InP/GaP/ZnS-QDs, showing features consistent with a mixture of InP and GaP domains rather than a uniform core–shell structure, InP reference pattern (red), and GaP reference pattern (blue); and (c) TEM image of InP/GaP-QDs.

**Figure 4:**
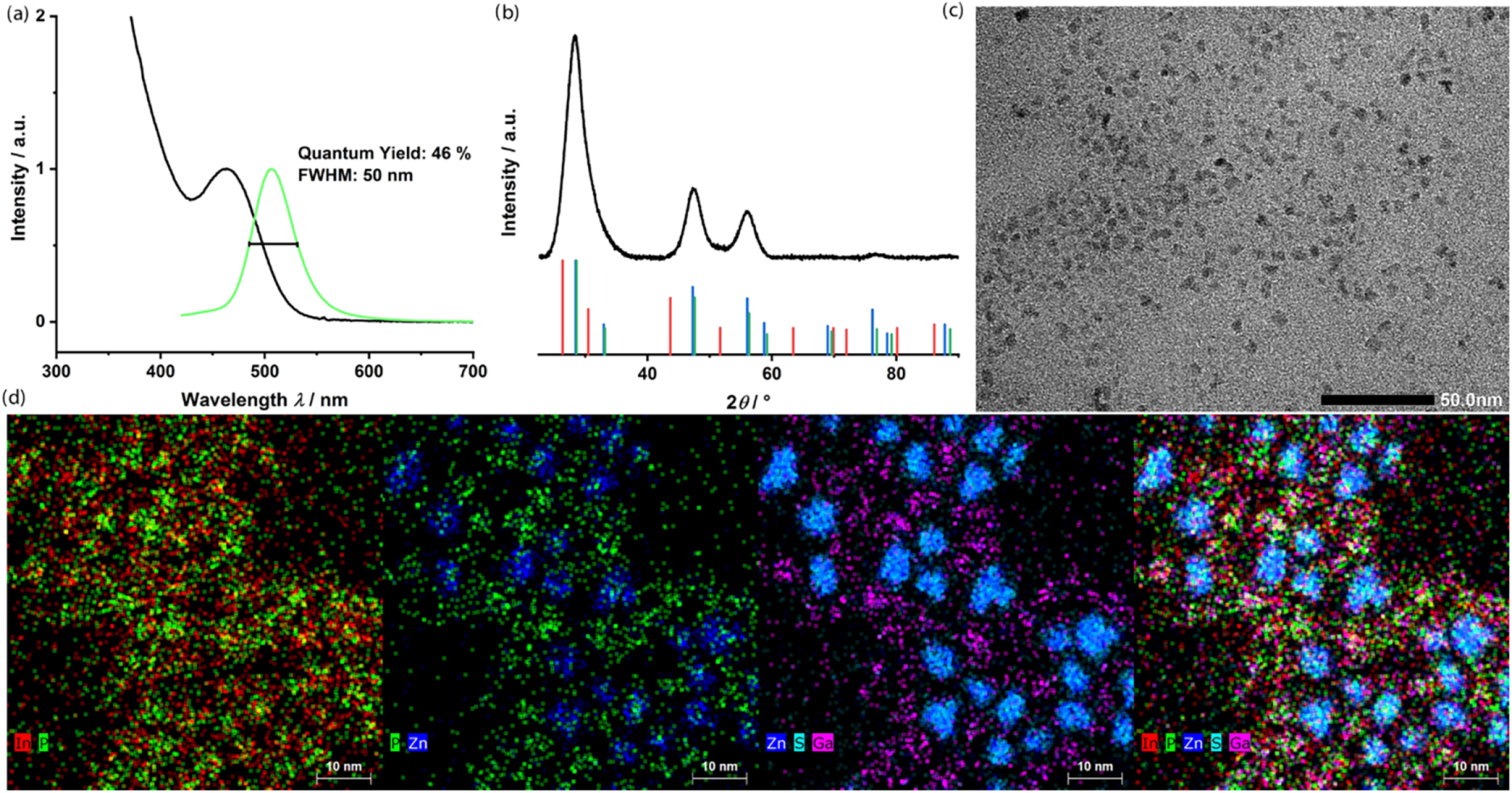
(a) UV/vis absorption and fluorescence spectra of InP/GaP/ZnS; (b) pXRD pattern of InP/GaP/ZnS-QDs, InP reference pattern (red), GaP reference pattern (blue), ZnS reference pattern (green); (c) TEM image of InP/GaP/ZnS-QDs; (d) EDXS-based element distributions reveal two distinct populations of particles, indicating a heterogeneous product composition.

Remarkably, with only slight optimizations of the synthesis procedures, the observed FWHM for InP/GaP/ZnS-QDs is among the narrowest reported in the literature. To the best of our knowledge, the FWHM for unprotected InP-QDs by synthesis with aminophosphanes belongs to the narrowest to date. This highlights the competitiveness of our newly developed method, even if the resulting product is a mixture of particle types rather than a single well-defined core/shell architecture and is not yet suited for optoelectronic applications. Here, control over the heterostructure synthesis must be improved. Both InP/GaP/ZnS and InP/ZnSeS/ZnS systems provide a strong foundation for further optimization, making them attractive and sustainable routes for InP-based QD synthesis, and this is the topic for ongoing investigations.

## Conclusions

In this study, we have successfully demonstrated the use of Rieke-indium (In*) as an efficient indium precursor and reducing agent for the synthesis of InP quantum dots (QDs) using aminophosphanes. By employing In*, we significantly reduced the required amount of aminophosphane, avoiding the usual disproportionation reaction of the phosphorus precursor. This led to minimized formation of phosphonium salt byproducts, thus enabling a more atom-efficient and environmentally benign synthetic route. To support this approach, we developed a novel oleylamine-free 0.4 M P­(OLA)_3_ stock solution in ODE, which proved compatible with our In* precursor. Our data highlight the excellent size tunability achievable with this system, as illustrated by using different zinc salts and tailoring P:In ratios. Notably, we synthesized green-emitting InP/GaP/ZnS QDs with a nonoptimized quantum yield (QY) of 46%, a narrow full width at half-maximum (FWHM) of 50 nm, and an emission maximum at 510 nm. Detailed structural analysis revealed the formation of a statistical mixture of InP- and GaP-rich particles rather than a distinct core–shell configuration. Despite this, the optical quality of the resulting QDs remains highly competitive.

Looking ahead, we believe that the current method, similar to P­(Me_3_Si)_3_-based approaches, can be further optimized to reduce the FWHM, improve emission wavelength control, and enhance quantum yields. By refining shelling procedures and improving particle homogeneity, we anticipate broader applicability of this In*-based strategy for the sustainable production of high-quality InP-based nanomaterials.

## Experimental Section

### Chemicals

InCl_3_ (99,99%, anhydrous), GaCl_3_ (99,999%, ultradry), ZnBr_2_ (98%, anhydrous), ZnCl_2_ (99,95%, anhydrous), Zn­(OAc)_2_ (99,9%, anhydrous), and trioctylphosphane (TOP, >97%) were purchased from ABCR. Zn­(stearate)_2_ (65%), potassium (98%), octadecene (ODE, 90%), sulfur (99,99%+, flakes), selenium (99,5%, 100 mesh), 3,5-dimethylpyrazole (99%), and TMSCl (>99%,) were obtained from Sigma-Aldrich. Myristic acid (>99%) and hexadecane (HD, >98%) were obtained from TCI Chemicals. Oleylamine (OLAH, 80–90%), ZnI_2_ (98%), and PCl_3_ (99%) were obtained from ACROS. Triethylamine, xylene, pentane, ethanol, dichloromethane, and toluene were purchased from Fisher Chemicals. Deuterated benzene, dichloromethane, and xylene were dried with CaH_2_ (CH_2_Cl_2_) or Na (C_6_D_6_) and distilled under inert gas prior to usage. ODE was degassed prior to usage. Oleylamine and PCl_3_ were distilled under a nitrogen atmosphere. Sulfur was sublimed prior to usage. All other chemicals were used as purchased and handled under inert conditions. All glassware was oven-dried at 160 °C prior to use. All reactions were conducted under Schlenk conditions.

### Synthesis

#### In* (0.2 M) in Xylene

InCl_3_ (2 mmol, 442.4 mg) and K(s) (6 mmol, 234.6 mg) were charged in a three neck flask equipped with a temperature sensor, a reflux condenser with bubbler, and a septum. The flask was evacuated at RT for 15 min before it was set under a N_2_ atmosphere. Then, 10 mL of dry xylene was added. The dispersion was reacted under reflux conditions at 140 °C for 6 h unless otherwise specified. A very fine, gray dispersion was obtained that precipitates after a few minutes upon discontinuation of stirring. The In* synthesis yielded the product in quantitative amounts. In* was stored under nitrogen atmosphere and typically consumed within 1–2 weeks. No long-term stability tests were conducted.

#### InP-Core Heat-Up Synthesis (Synthesis Procedure 1)

Zn­(OAc)_2_ (0.4 mmol, 73.4 mg) and myristic acid (0,8 mmol, 182.7 mg) were suspended in ODE (4 mL) and flushed with N_2_ and evacuated three times for 2 min at 120 °C to remove acetic acid. The solution commenced degassing for 1h at this temperature. Typically, *p* < 10^–3^ mbar were reached. Afterward, the as-synthesized In* in xylene (0.2 M, 1 mL) stock solution was added and the dispersion was degassed for 5 min at 120 °C. Typically *p* = 5 × 10^–2^ mbar were reached. Subsequently, the mixture was left to cool down to room temperature. P­(OLA)_3_ (0.4 M in ODE, 0.25 mL) was added and the mixture was then heated to 200 °C with a heating rate of 35 °C/min. The mixture was left to stir for 30 min. Purification was performed by one precipitation/redispersion cycle using ethanol and toluene as nonsolvent and solvent during centrifugation, respectively. Precipitation was performed by adding ethanol in a 1:1 volume ratio. Redispersion was achieved by dissolving the QDs in 3 mL of toluene. When using octadecene, an oily product was obtained at the end of purification. The final precipitate was redispersed in toluene and used for further characterization.

#### InP-Core Heat-Up Synthesis (Synthesis Procedure 1a) −QD Preparaion for pXRD and TEM

Zn­(OAc)_2_ (0.4 mmol, 73.4 mg) and myristic acid (0,8 mmol, 182.7 mg) were suspended in hexadecane (4 mL) and flushed with N_2_ and evacuated three times for 2 min at 120 °C to remove acetic acid. The solution commenced degassing for 1h at this temperature. Typically, *p* < 10^–3^ mbar were reached. Afterward, the as-synthesized In* in xylene (0.2 M, 1 mL) stock solution was added and the dispersion was degassed for 5 min at 120 °C. Typically *p* = 5 × 10^–2^ mbar were reached. Subsequently, the mixture was left to cool to room temperature. P­(OLA)_3_ (0.4 M in hexadecane, 0.25 mL) was added and the mixture was then heated to 200 °C with a heating rate of 35 °C/min. The mixture was left to stir for 30 min. Purification was performed by three precipitation/redispersion cycles using ethanol and toluene as nonsolvent and solvent during centrifugation, respectively. Precipitation was performed by adding ethanol in a 1:1 volume ratio. Redispersion was achieved by dissolving the QDs in 3 mL of toluene.

#### InP-Core Hot-Injection Synthesis (Synthesis Procedure 2)

ZnX_2_ (0.4 mmol) was suspended in ODE (4 mL) and degassed for 1 h at 120 °C. In case of Zn­(myristate)_2_, the salt was generated in situ from Zn­(OAc)_2_ and myristic acid during this period. Afterward, the as-synthesized In* in xylene (0.2 M, 1 mL) stock solution was added and the dispersion was degassed for 5 min at 120 °C. Typically p = 5 × 10^–2^ mbar were reached. The mixture was then heated to 200 °C, where P­(OLA)_3_ (0.4 M in ODE, 0.25 mL) was rapidly injected. The mixture was left to stir for 30 min. Purification was performed by one precipitation/redispersion cycle using ethanol and toluene as nonsolvent and solvent during centrifugation, respectively. Precipitation was performed by adding ethanol in a 1:1 volume ratio. Redispersion was achieved by dissolving the QDs in 3 mL of toluene. The final precipitate was redispersed in toluene and used for further characterization.

#### InP/ZnS Synthesis

After the InP-core synthesis according to synthesis procedure 2, instead of cooling down to room temperature, zinc diethyldithiocarbamate (0.2 equiv dispersed in ODE (0.1 M)) was rapidly injected. The mixture was heated to 230 °C, where the reaction was left to continue for 1 h. Afterward, the reaction mixture was cooled to room temperature. Purification was performed by one precipitation/redispersion cycle using ethanol and toluene as nonsolvent and solvent during centrifugation, respectively. Precipitation was performed by adding ethanol in a 1:1 volume ratio. Redispersion was achieved by dissolving the QDs in 3 mL of toluene.

#### InP/GaP/ZnS-QD Synthesis

Zn­(OAc)_2_ (0.4 mmol, 73.4 mg) and myristic acid (0.8 mmol, 182.7 mg) were suspended in ODE (4 mL) and flushed with N_2_ and evacuated thrice at 120 °C to remove acetic acid. The solution commenced degassing for 1h at given temperature. Typically *p* < 10^–3^ mbar were reached. Afterward, the as-synthesized In* in xylene (0.2 M, 1 mL) stock solution was added and the dispersion was degassed for 5 min at 120 °C. Typically *p* = 5 × 10^–2^ mbar were reached. Subsequently, the mixture was left to cool down to room temperature, where GaCl_3_ (0.071 M in ODE, 1 mL) and P­(OLA)_3_ (0.4 M in ODE, 0.25 mL) were added. The mixture was heated to 200 °C at a heating rate of 35 °C/min. At 200 °C, the mixture was stirred for 10 min. Afterward, TOP-S (0.15 mL, 2.24 M, 0.336 mmol) was added, and the mixture was stirred for a further 30 min. Zn­(stearate)_2_ stock solution (0.66 mL, 1 g in 4 mL ODE) was added and the temperature was increased to 220 °C for 30 min. Then, TOP-S (0.1, 2.24 mL, 0.224 mmol) was added, and the shelling proceeded for 30 min at 240 °C before injecting the second Zn­(stearate)_2_ partition (0.34 mL, 0.5 g in 2 mL of ODE) and heating to 260 °C for 30 min. After cooling to room temperature, the InP/GaP/ZnS-QDs were purified by three precipitation/redispersion cycles using ethanol and toluene as nonsolvent and solvent during centrifugation, respectively. Precipitation was performed by adding ethanol in a 1:1 volume ratio. Redispersion was achieved by dissolving the QDs in 3 mL of toluene. For optical characterization, samples from reactions conducted in ODE were used. In case of characterization by pXRD, TEM, or HAADF-STEM, the samples of reactions performed in hexadecane were used. When using octadecene, an oily product was obtained at the end of purification. The final precipitate was redispersed in toluene and used for further characterization. Note that STEM-EDXS revealed a mixture of Ga-rich and Zn-rich particles, rather than a distinct core–shell architecture.

#### InP/ZnSeS/ZnS-QD Synthesis

ZnBr_2_ (0.9 mmol, 202.7 mg) was suspended in ODE (5 mL) and OLAH (0.5 mL) and degassed for 1 h at 120 °C. Afterward, the as-synthesized In* in xylene stock solution (0.2 M, 2.25 mL) was added and the dispersion was degassed for 5 min at 120 °C. Typically 5 × 10^–2^ mbar were reached. The mixture was then heated to 200 °C, where P­(OLA)_3_ in OLAH (0.5 mL, 0.9 mL) was rapidly injected. The reaction was left to proceed for 7 min at 180 °C before adding TOP-Se in TOP (0.12 M, 0.5 mL) and heating to 200 °C for 30 min. Then, Zn­(stearate)_2_ in ODE (0.375 g in 1.5 mL of ODE) was added, the temperature was increased to 220 °C, and the reaction was left to commence for 30 min. Starting from the second cycle, TOP-S was also added. In total, three cycles of TOP-Se (0.25 and 0.09 mL respectively, 0.12 M), TOP-S (0.46 and 0.91 mL respectively, 2.24 M), and Zn­(stearate)_2_ in ODE (0.375 g in 1.5 and 0.375 g in 1.5 mL respectively) were injected. At the end, the reaction was kept for 1 h at 300 °C before cooling to 220 °C. Then, n-DDT (0.75 mL) was added and the reaction was stirred for 30 min. Three more cycles of n-DDT addition were performed (0.75 mL, 30 min at 220 °C; 0.5 mL, 30 min at 230 °C 0.5 mL, 1 h at 240 °C, respectively). Then, the reaction mixture was cooled to 190 °C before zinc acetate (375 mg, 1.5 mmol) was added in a N_2_ flow and left to stir for 2 h. After completion, the reaction was left to cool to room temperature. Purification was performed by one precipitation/redispersion cycle using ethanol and toluene as nonsolvent and solvent during centrifugation, respectively.

**P­(OLA)**_**3**_ **in OLAH (0.5 M)** were synthesized according to previously reported literature procedures.^23^

#### P­(OLA)_3_ in ODE (0.4 M)

The reaction was performed analogously to the reaction in oleylamine. Tris­(3,5-dimethylpyrazolyl)­phosphane (5 mmol, 1.52 g), ODE (10 mL) and OLAH (5.5 mL) were charged into a 100 mL Schlenk tube fitted with a cooling finger. The mixture was stirred for 1 h at 80 °C under vacuum to remove the resulting pyrazole. If needed, the resublimation step was repeated, until <1% of starting material and pyrazole remained according to NMR-analysis.

#### TOP-Se (0.12 M)

Selenium (189.5 mg) was dissolved under mild stirring over 24 h in TOP (20 mL) at room temperature inside a glovebox.

#### TOP-S (2.24 M)

Sulfur (1.436 g) was dissolved under mild stirring over 24 h in TOP (20 mL) at room temperature inside a glovebox.

#### Zn­(stearate)_2_ in ODE

Zn­(stearate)_2_ (4g) was degassed under mild stirring for 1h. Then, ODE (16 mL) was added in a N_2_ atmosphere. The dispersion was heated to 140 °C under vacuum for 2 h before cooling down and storing the viscous, fine dispersion under a N_2_ atmosphere until use.

### Characterization

**Nuclear magnetic resonance (NMR)** spectra were measured on a Bruker AVANCE III HD Nanobay, 400 MHz UltraSield (^1^H (400.13 MHz), ^13^C (100.61 MHz), ^31^P (161.98 MHz)) or on a Bruker AVANCE III HDX, 500 MHz Ascend (^1^H (500.13 MHz), ^13^C (125.75 MHz), ^31^P (202.45 MHz)) spectrometer. All ^13^C NMR spectra were exclusively recorded with composite pulse decoupling. Chemical shifts were referenced to δ­(TMS) = 0.00 ppm (^1^H, ^13^C) and δ­(H_3_PO_4_, 85%) = 0.00 ppm (^31^P, externally). Chemical shifts (δ) are reported in ppm. Coupling constants (*J*) are reported in Hz. NMR spectra were analyzed by using the Topspin program.

**UV/Vis Spectroscopy.**The absorption spectra of QD solutions were measured on a PerkinElmer Lambda 2 ultraviolet–visible spectrometer. Standard or optical characterization, samples from reactions conductedard *d* = 1 cm quartz cuvettes, and toluene as solvent were used.

**Fluorescence spectroscopy** was performed by a Fluoromax-4 spectrofluorometer (Horiba Jobin Yvon Inc.) equipped with a PMT detector for the visible range to acquire the steady-state PL spectra of solutions of InP/GaP/ZnS-QDs and for FWHM estimations.

**Absolute photoluminescence quantum yield (QY)** measurements were performed by using a FluoroLog-3 spectrofluorometer (Horiba Jobin Yvon) equipped with a Quanta-φ integrating sphere. QDs in solution were investigated in 10 mm × 4 mm quartz cuvettes. The absorbance at the excitation wavelength was adjusted between 0.05–0.1 prior to the measurement.

**Powder X-ray diffraction (pXRD)** patterns were collected in reflection mode with a Bruker AXS D2 PHASER diffraction system using Cu K-α irradiation (λ = 1.5406 Å) and a LYNXEYE/SSD160 detector.

**Transmission electron microscopy (TEM)** of the QDs was performed by using a FEI Tecnai F30 microscope operated at 300 kV.

**High-angle annular dark-field scanning transmission electron microscopy (HAADF-STEM)** imaging and spectrum imaging analysis based on energy-dispersive X-ray spectroscopy (EDXS) were performed at 200 kV with a Talos F200X microscope equipped with an X-FEG electron source and a Super-X EDXS detector system (FEI). Prior to STEM analysis, the specimen mounted in a high-visibility, low-background holder was placed for 2 s into a Model 1020 Plasma Cleaner (Fischione) to remove potential organic contamination. Since, during EDXS-based element mapping, the electron beam interacted strongly with the TEM specimens leading to relatively fast degeneration of the latter ones, it was not possible to obtain reliable data for the pure InP-core QDs. In the case of the InP/GaP/ZnS QDs with heterogeneous composition, the data recording time was limited to find an acceptable trade-off between QD stability and EDXS counting statistics.

## Supplementary Material



## Notes

Electrochemical methods CV and SCW spectra, UV/vis absorption and fluorescence spectra, NMR spectra, particle size distribution and cost table (PDF)
