# Supplementary material for: Sustainable Synthesis of InP Quantum Dots via Rieke-Indium Reduction: Size Control and Ensemble Optical Properties
Source: Inorg Chem. 2025 Aug 18;64(34):17200–9. doi: 10.1021/acs.inorgchem.5c02060 (PMC12406191; doi:10.1021/acs.inorgchem.5c02060)
Supplement: Supplementary file 1 [file ic5c02060_si_001.pdf]

Supporting Information

Sustainable Synthesis of InP Quantum Dots via Rieke-Indium Reduction: Size Control and Ensemble Optical Properties

Michael A. Müller,[a] Stephen Schulz,[a] Kai Schwedtmann,[a] Thorben Starzynski,[b] René Hübner,[c] Alexander Eychmüller,[b] Jan J. Weigand[a,*]

[a] Chair of Inorganic Molecular chemistry; [b] Chair of Physical Chemistry - Faculty of Chemistry and Food Chemistry, Technical University Dresden, 01062 Dresden, Germany [c] Institute of Ion Beam Physics and Materials Research, Helmholtz-Zentrum Dresden-Rossendorf, Bautzner Landstrasse 400, 01328 Dresden.

Prof. Jan J. Weigand*

jan.weigand@tu-dresden.de

Table of Contents

1. Electrochemical characterization of $P(OLA)_3$ s2
2. UV/Vis spectra of InP- and InP/ZnS-QDs solutions synthesized by using different In:P ratios S3
3. ^{31}P NMR spectra of reaction mixtures of In* with $P(OLA)_3$ at 200°C in ODE and OLAH S4
4. InP- and InP/ZnS-QDs with and without methanol washing of In* S5
5. Diagram of FWHM (emission data) and emission maxima of InP/ZnS-QDs solutions in toluene S5
6. Estimation on savings of production cost S6
7. Fluorescence spectra of orange-emitting InP and InP/ZnS-QDs S6
8. UV/Vis absorbance and Fluorescence spectra of InP/ZnS-QDs S7
9. Particle Size distribution of InP/GaP/ZnS-QDs S7
10. UV/Vis absorbance and Fluorescence Spectra of multiple InP/GaP/ZnS-QDs Syntheses S8
11. UV/Vis absorbance and Fluorescence Spectra of InP/ZnSeS/ZnS-QDs S8

1. Electrochemical characterization of $P(OLA)_3$

All electrochemical (*EC*) measurements were performed in a Glovebox Pure Lab HE GP-1 SR (Innovative Technology, USA) in an atmosphere of purified nitrogen (<0.1ppm O_2; <0.1ppm H_2O). The glovebox was equipped with military grade BNC feedthroughs in a homemade gas tight flange for low noise electrical connection of electrochemical cells inside. EC cells were connected to a PGSTAT302 (Metrohm Autolab, Utrecht, The Netherlands) E = ±10 V, U = ±35 V with an auxiliary voltage monitor (BK Precision 2831E, Yorba Linda, CA USA). NOVA Software (Metrohm Autolab) Version 1.11.2 was used to control the potentiostat and magnetic stirring. Data analysis of electrochemical data was performed using OriginPro 2019 (OriginLab Cooperation, Northampton, MA, USA).

Solvent preparation / supporting electrolyte preparation

CH_2Cl_2 was dried by heating with CaH_2 to reflux conditions for at least 6 h and distillation under Argon or reduced pressure (DMSO) onto vacuum activated molecular sieves (CH_3CN: 3 Å ; DMSO: 4 Å, $1\cdot10^{-3}$ mbar, 350 °C, 12 h). Additionally all solvents were stored over vacuum activated molecular sieves for at least 14 days. [^{n}Bu$_4$N][OTf] was dried at least 5 times by dissolving in CH_2Cl_2, evaporating the solvent to high vacuum at 80 °C and finally drying in high vacuum ($1\cdot10^{-3}$ mbar) for 2 days at 140 °C. Prior to each measurement the solvent is passed through a Pasteur pipette with an activated ($1\cdot10^{-3}$ mbar, 350 °C, 24 h) aluminum oxide bed (*D* = 5 mm; *L* = 70 mm) in the glove box before the supporting electrolyte is added.

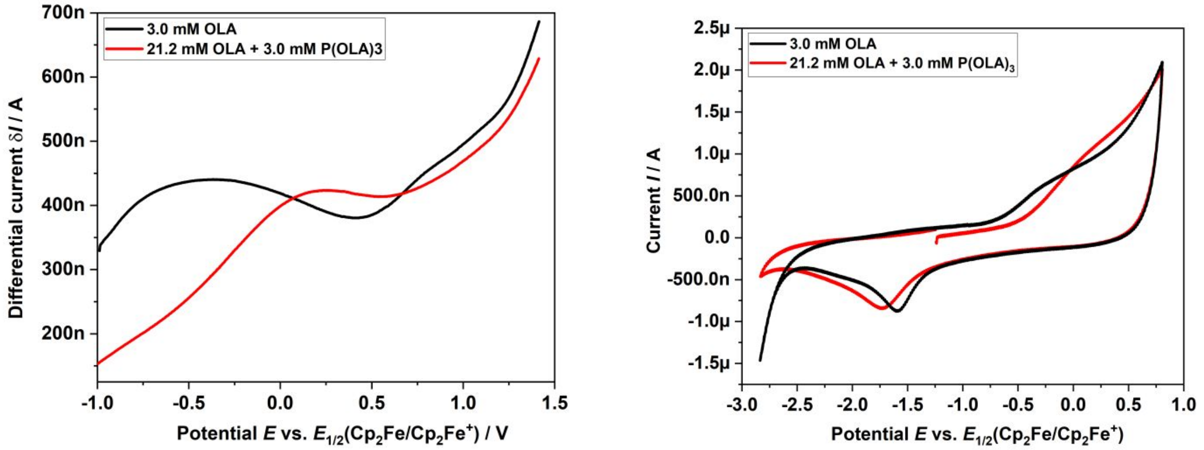

Figure S1: Left: Square wave voltammogram (SWV), right: cyclic voltammogram (CV) of a $P(OLA)_3$ (3.0 mM) in 0.1 M [^{n}Bu$_4$N][OTf] electrolyte solution in presence of 21.2mM oleylamine at a 3 mm platinum disc electrode measured in CH_2Cl_2 with a scan rate of v = 0.063 V/s for SWV and v = 1.0 V/s for CV. Half-wave potentials of OLAH ("$E_{1/2}$" = 0.17 V) in comparison of $P(OLA)_3$ in OLAH ("$E_{1/2}$" = -0.45V) are deteminated referenced to the half-wave potential of the redox-pair Cp_2Fe/Cp_2Fe^+. Electroanalytical measurements in ODE were not possible due to the poor solubility of supporting electrolytes in ODE.

2. UV/Vis spectra of InP- and InP/ZnS-QDs solutions synthesized by using different In:P ratios

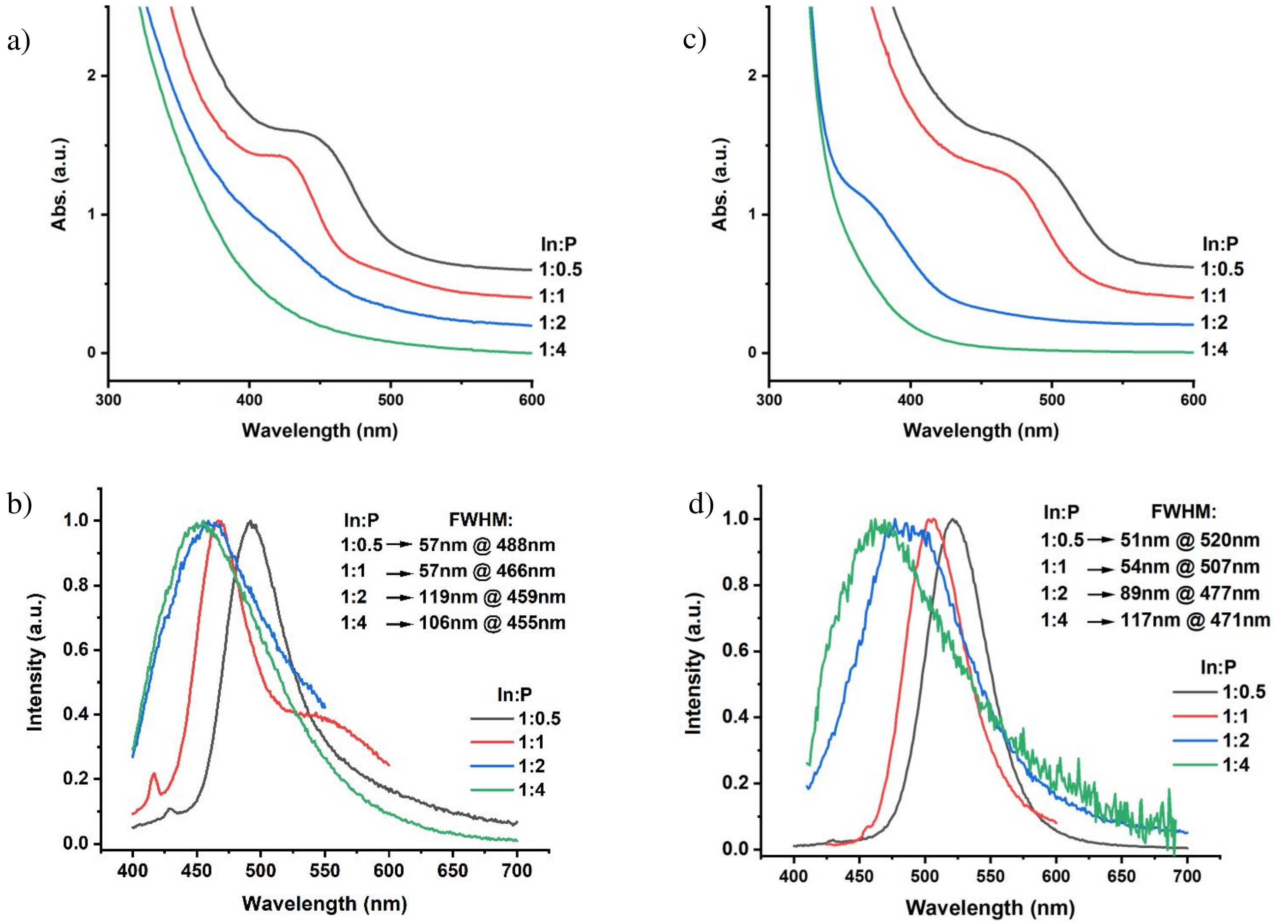

Figure S2: UV/Vis absorption and fluorescence spectra with corresponding FWHM values for different In:P ratios for a) and b) InP-cores and c) and d) InP/ZnS-QDs respectively. Increasing the amount of $P(OLA)_3$ leads to a pronounced increase in FWHM and a blue-shift in the emission maxima, consistent with enhanced nucleation and restricted growth as described in the manuscript.

3. ^{31}P NMR spectra of reaction mixtures of In* with $P(OLA)_3$ at 200°C in ODE and OLAH

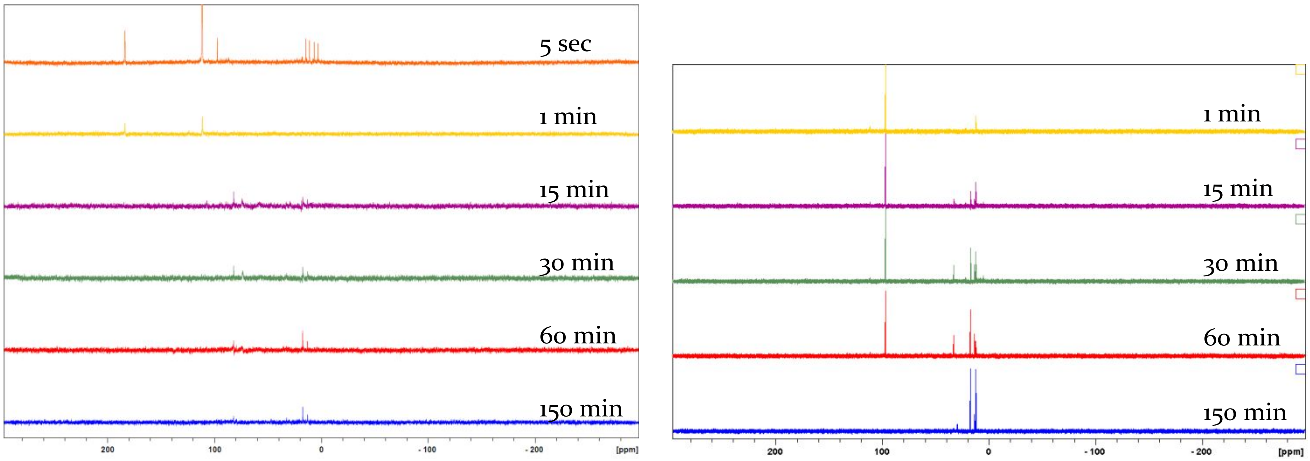

Figure S3: ^{31}P-NMR monitoring of samples of the InP-QD formation reaction in ODE as solvent (left side) at 200 °C over 150 min (C_6D_6 capillary, 85% H_3PO_4) and in OLAH as solvent (right side) over 150 min (C_6D_6 capillary, 85% H_3PO_4). The reactions were conducted according to synthesis procedure 2 and are consequently hot-injection reactions. Reaction times describe the time past $P(OLA)_3$ injection into the reaction mixture at 200°C, not when the reaction mixture returned to 200°C. The syringe used for taking the reaction aliquote contained 0.4 ml dry toluene. Upon take up of the reaction mixture, the temperature was lowered immediately beneath the necessary temperature for core growth. The reaction in ODE leads to an almost complete consumption of $P(OLA)_3$ and its derivatives (δ = 111.91 ppm) within minutes. No phosphonium salt $[(POLA)_4]$[myristate] is formed due to the disproportionation reaction of $P(OLA)_3$ that typically occurs during aminophosphane based syntheses of InP-QDs. Meanwhile, the reaction of In* with $P(OLA)_3$ in OLAH (δ = 96.95ppm) leads to the partial conversion to phosphonium salt $[(POLA)_4]$[myristate] (δ = 33.04 ppm). Afterwards, $[(POLA)_4]$[myristate] is converted to other unknown side products.

Reaction in ODE		Reaction in OLAH	
Chemical Shift δ	Compound	Chemical Shift δ	Compound
183.5	$P_2(OLA)_4$	111.5	$P_2(OLA)_5$
111.9	$P_2(OLA)_5$	97.0	$P(OLA)_3$
97.4	$P(OLA)_3$	33.1	$[P(OLA)_4]$[Myristate]
82.1	unknown - trace amount	17.3	unknown
79.6	unknown - trace amount	13.7	unknown
17.4	unknown - trace amount		
13.7	unknown - trace amount		

4. InP- and InP/ZnS-QDs with and without methanol washing of In*

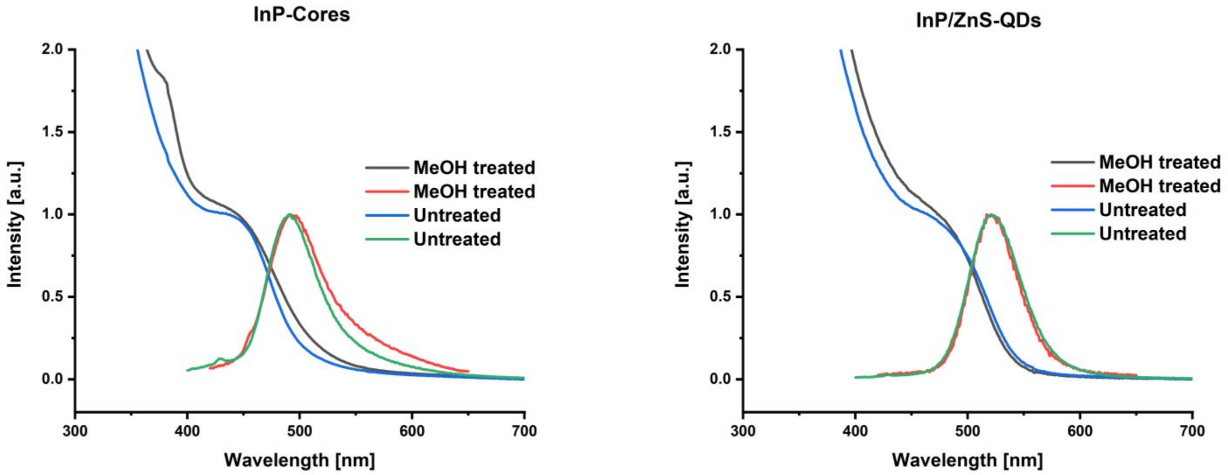

Figure S4: UV/Vis and fluorescence spectra of InP-core (left) and InP/ZnS (right) QDs synthesized from MeOH-washed and unwashed In*. Methanol washing removes KCl but results in broader emission for InP-core QDs (FWHM: 59 nm vs. 47 nm) and slightly narrower emission for shelled InP/ZnS QDs (FWHM: 49 nm vs. 51 nm), potentially due to altered surface chemistry.

5. Diagram of FWHM (emission data) and emission maxima of InP/ZnS-QDs solutions in toluene synthesized by using In* for different reaction durations

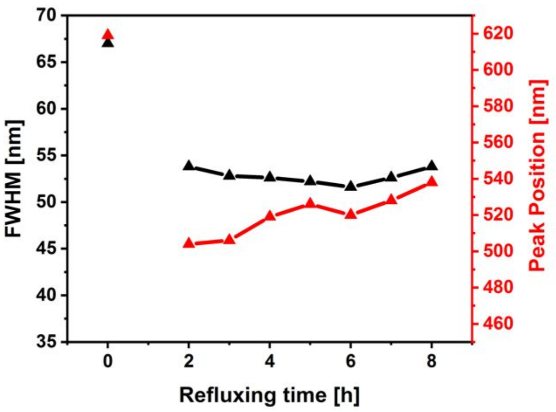

Figure S5: Diagram showing emission properties of InP/ZnS-QD in toluene solutions. For each set of FWHM and emission maxima, the QD syntheses used In* which has been refluxing for different durations. When the reflux time of In* was 1 h, no fluorescence was observed after conducting the InP/ZnS-QDs synthesis. For the data point at 0 hours of In* synthesis time, the In* reaction set-up was performed as usual but instead of heating the reaction mixture of $InCl_3$, potassium and xylene to the refluxing temperature, the mixture was stirred for 5 minutes at room temperature. Then the mixture was used like the In*-dispersion in the synthesis of InP-QDs.

6. Estimation on savings of production cost

The price for 1g (6.86mmol) of InP-QDs (P:In = 1:1) is calculated. Prices of chemicals (largest batches available for lab purposes):

$InCl_3$ (99.99%, 250g, abcr): 695.8€; K (99.8% purity, 1kg): 229€; Xylene (mixture, >75%, 1l, Sigma Aldrich): 35,9€ ; $P(NMe_2)_3$ (97%, 250g, Alfa Aesar): 644€; $ZnCl_2$ (99.95%, 100g, ABCR): 180€; OLAH (80-90% purity, 5l, Acros): 501€; $Zn(OAc)_2$ (99.9% purity, 250g, abcr); 352€; Myristic Acid (99% purity, 500g, TCI Chemicals): 26€; ODE (90% purity, 2.5l, Acros): 89.9€.

In*-route				$InCl_3$-route			
Chemical	Mole	Weight or Volume	Price	Chemical	Mole	Weight or Volume	Price
$InCl_3$	13.72 mmol	1.517 g	8.44€				
K	20.56 mmol	0.804 g	0.18€				
Xylene		34.3 ml	1.23€				
$P(NMe_2)_3$	6.86 mmol	1.697g	4.37€				
OLAH	20.58 mmol	6.8 ml	0.68€	$InCl_3$	6.86 mmol	1.517 g	4.22€
$Zn(Oac)_2$	13.72 mmol	3.012 g	4.24€	$P(NMe_2)_3$	27.44 mmol	6.788 g	17.49€
Myristic Acid	27.44 mmol	6.266 g	0.33€	$ZnCl_2$	34.3 mmol	4.674 g	8.41€
ODE		137.2 ml	4.93€	OLAH		171.5 ml	17.18€
Total:	24.4€ for 1g of InP			Total:	47.3€ for 1g of InP		

Table S1: In total (47.3€/g-23.7€/g)/47.3€/g), costs can be reduced by approximately 50 % due to reduction of $P(NMe_2)_3$ use.; Full conversion of $P(NMe_2)_3$ and simple scale up is assumed for both routes. Energy costs are excluded. Costs for work up and waste treatment are excluded. Potential cost savings due to Indium recovery or due to higher than 1:1 (In:P) incorporation are excluded. For easier comparison and since $P(OLA)_3$ is not commercially available, $P(NMe_2)_3$ is taken as P-precursor in both syntheses. Date of data acquisition: 10/22/2021. Further savings arise from reduced waste disposal costs.

7. Fluorescence spectra of orange-emitting InP and InP/ZnS-QDs

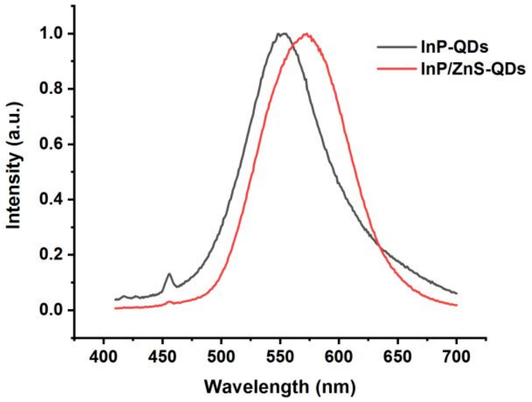

Figure S6: Fluorescence spectra of InP and InP/ZnS QDs synthesized with $ZnCl_2$, exhibiting orange emission with FWHMs of 81 nm and 88 nm at emission maxima of 553 nm and 573 nm, respectively. The red-shifted emission reflects the influence of heavier halide zinc salts on QD growth and optical properties.

8. UV/Vis absorbance and Fluorescence spectra of InP/ZnS-QDs

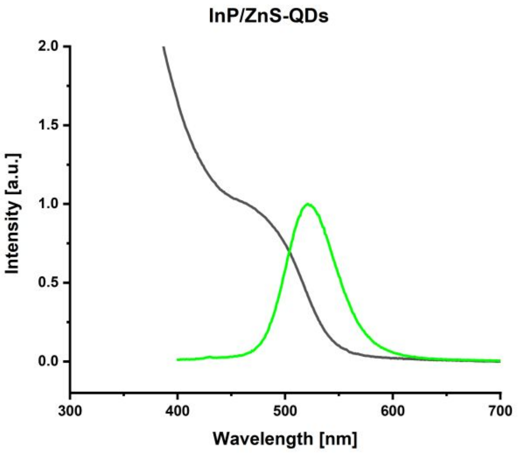

Figure S7: UV/Vis absorption and fluorescence spectra of InP/ZnS QDs obtained via the In* route. The QDs exhibit a FWHM of 51 nm and an emission maximum at 520 nm. Despite the relatively narrow emission profile, the quantum yield remains below 10%, as noted in the manuscript.

9. Particle Size distribution of InP/GaP/ZnS-QDs

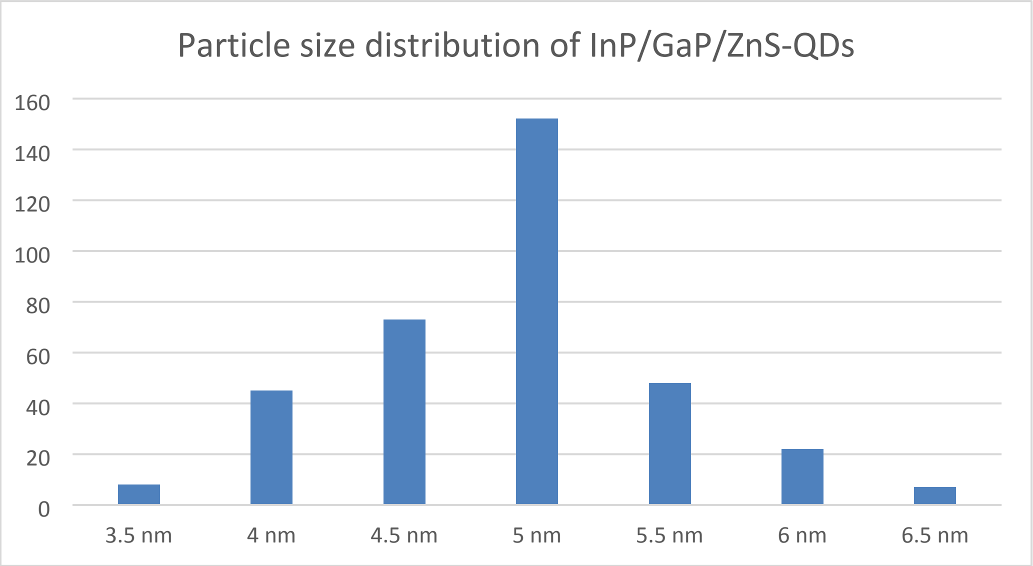

Figure S8: Histogram of particle sizes for InP/GaP/ZnS QDs based on TEM image analysis (n = 355). The size distribution yields an average particle diameter of 4.9 ± 1.2 nm (two standard deviations). The data reflects a heterogeneous ensemble comprising both Ga-rich and Zn-rich QD populations, rather than a uniform core–shell architecture.

10. UV/Vis absorbance and Fluorescence Spectra of multiple InP/GaP/ZnS-QDs Syntheses

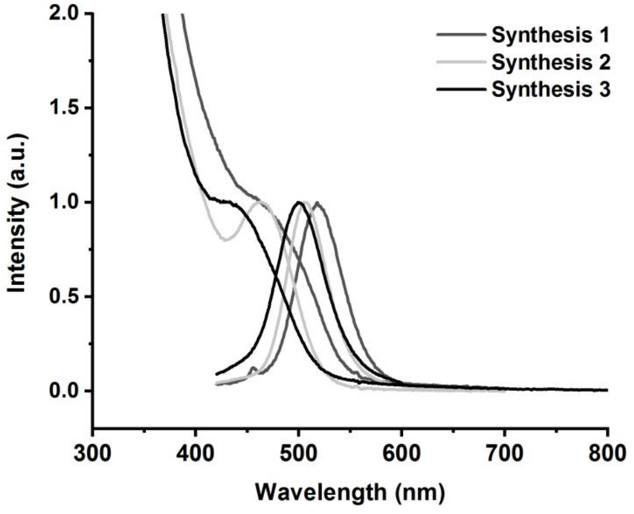

Figure S9: UV/Vis absorption and fluorescence spectra of three independent syntheses of InP/GaP/ZnS QDs, demonstrating good reproducibility. Emission maxima were observed at 518 nm (FWHM 53 nm), 510 nm (FWHM 50 nm), and 500 nm (FWHM 56 nm), respectively. Hence emission maxima with a peak position of on average 509 nm +/- 10 nm were obtained, when using the same synthesis procedure. Meanwhile, FWHM of 53nm +/- 3 nm were obtained. Further refinement of the distribution could be obtained by increasing the amount of conducted reactions. Variation for quantum yield has not been determined. These optical signatures correspond to a statistical mixture of QD species, not discrete core–shell structures.

11. UV/Vis absorbance and Fluorescence Spectra of InP/ZnSeS/ZnS-QDs

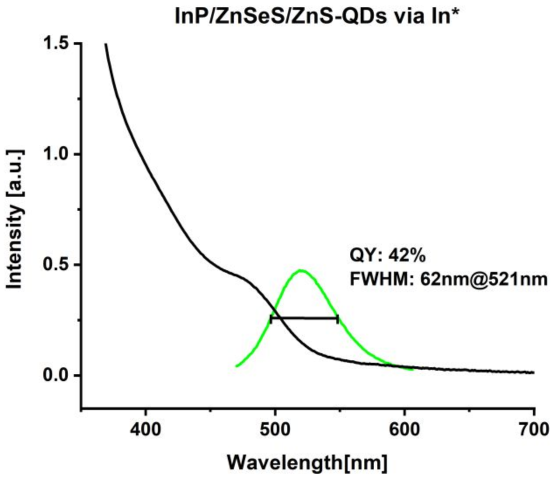

Figure S10: UV/Vis absorption and fluorescence spectra of InP/ZnSeS/ZnS-QDs synthesized using In*. A FWHM of 62 nm and a quantum yield of 42% were observed.
